# A Ratiometric Fluorescent Sensor for Cd^2+^ Based on Internal Charge Transfer

**DOI:** 10.3390/s17112517

**Published:** 2017-11-02

**Authors:** Dandan Cheng, Xingliang Liu, Yadian Xie, Haitang Lv, Zhaoqian Wang, Hongzhi Yang, Aixia Han, Xiaomei Yang, Ling Zang

**Affiliations:** 1 Chemical Engineering College, Qinghai University, Xining 810016, China; 1994990022@qhu.edu.cn (D.C.); liuxingliang@qhu.edu.cn (X.L.); 1991990011@qhu.edu.cn (Y.X.); 1989990029@qhu.edu.cn (H.L.); 1990990009@qhu.edu.cn (Z.W.); yhz17@mails.tsinghua.edu.cn (H.Y.); 2 Department of Materials Science and Engineering, University of Utah, Salt Lake City, UT 84108, USA; jaimee@eng.utah.edu

**Keywords:** ratiometric fluorescent sensor, Me_4_BOPHY, Cd^2+^ ion, ICT

## Abstract

This work reports on a novel fluorescent sensor **1** for Cd^2+^ ion based on the fluorophore of tetramethyl substituted bis(difluoroboron)-1,2-bis[(1*H*-pyrrol-2-yl)methylene]hydrazine (Me_4_BOPHY), which is modified with an electron donor moiety of *N*,*N*-bis(pyridin-2-ylmethyl)benzenamine. Sensor **1** has absorption and emission in visible region, at 550 nm and 675 nm, respectively. The long wavelength spectral response makes it easier to fabricate the fluorescence detector. The sensor mechanism is based on the tunable internal charge transfer (ICT) transition of molecule **1**. Binding of Cd^2+^ ion quenches the ICT transition, but turns on the π − π transition of the fluorophore, thus enabling ratiometric fluorescence sensing. The limit of detection (LOD) was projected down to 0.77 ppb, which is far below the safety value (3 ppb) set for drinking water by World Health Organization. The sensor also demonstrates a high selectivity towards Cd^2+^ in comparison to other interferent metal ions.

## 1. Introduction

**Scheme 1:**
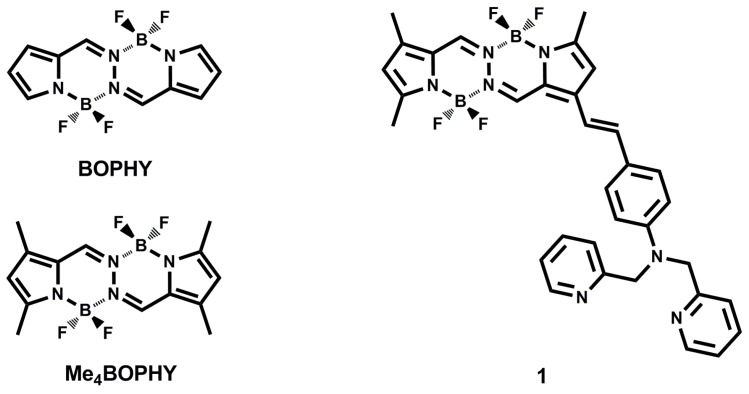
Molecule structures of bis(difluoroboron)-1,2-bis[(1*H*-pyrrol-2-yl)methylene]hydrazine (BOPHY), tetramethyl substituted BOPHY (Me_4_BOPHY) and sensor **1.**

Cadmium represents a highly toxic industrial and environmental pollutant, and it is classified as a human carcinogen. Exposure to cadmium may cause cancer mutation of some organs, such as lung, endometria, prostate, kidney, etc. [1]. World Health Organization (WHO) underlines drinking water value for cadmium as 3 ppb [2]. So, detection of cadmium at trace level remains an important task, for which cadmium ion (Cd^2+^) usually remains as the target for chemical sensors to monitor the cadmium pollution in water environment. Current methods for Cd^2+^ detection include UV-Vis spectrometry [3], atomic absorption spectrometry (AAS) [4], inductively coupled plasma atomic emission spectroscopy (ICP-AES) [5], and fluorescent sensors [6,7,8,9,10,11,12,13,14,15,16]. Among these, fluorescent sensors are uniquely compelling due to their high sensitivity, good selectivity [6,7,8,9,10,11,12,13,14,15,16], and capability for ratiometric sensing to further improve the detection sensitivity [17,18,19,20]. However, many fluorescence sensors for Cd^2+^ ion reported thus far have some technical drawbacks, for example, a poor limit of detection (LOD) [7,8,18], complicated synthesis of sensor molecules [6], solvent toxicity [7], and a hardly controlled fluorescence change [14]. In order to develop high performance fluorescent sensors, the fluorophore must be designed with both high quantum efficiency and chemical tunability in response to metal binding [21,22]. Borondipyrromethene (BODIPY) has long been studied as an outstanding organoboron fluorophore and been used in the development of fluorescent sensors for many metal ions [23,24,25,26,27], including Cd^2+^ ion [28,29]. In 2014, a novel organoboron compound bis(difluoroboron)-1,2-bis[(1*H*-pyrrol-2-yl)methylene]hydrazine (BOPHY) (Scheme 1) was reported [30,31,32]. BOPHY has distinctive absorption and emission features that are suited for sensor development, particularly when compared to those with spectral response in high energy blue or UV region. Many BOPHY derivatives have ever since synthesized [33,34,35], including several from our lab (F-BOPHY1-3) [36], which all showed high efficiency of fluorescence.

We noticed that there have been only two BOPHY fluorescent sensors reported so far, which were used for detecting Cu^2+^ and H^+^, respectively [37,38]. In this paper, we report on synthesis of a novel fluorescent sensor 1 for Cd^2+^ (Scheme 1) based on a BOPHY fluorophore substituted by tetramethyl group (Me4BOPHY), in conjugation through a vinyl link with an electron donor moiety *N*,*N*-bis(pyridin-2-ylmethyl)benzenamine (BPA). BPA is also a strong chelator to Cd^2+^ ion, thus affording high sensing sensitivity. Pristine sensor 1 exhibits a significant internal charge transfer (ICT) transition between Me4BOPHY and BPA, with an absorption and fluorescence extending into long wavelength, 550 nm and 675 nm, respectively. When chelated with Cd^2+^ the electron-donating power of BPA will be reduced, thus quenching the ICT transition and turning on the π − π transition of the fluorophore, which combined the results in blue-shift of the absorption and fluorescence of 1. Such dramatic spectral change can be used to develop efficient fluorescence sensor for Cd^2+^ detection, particularly through the ratiometric fluorescence modulation [39,40,41,42,43].

## 2. Experimental Methods

### 2.1. Materials and Instrumentation

All of the solvents and chemicals were purchased in analytical grade and were used as received. Column chromatography used 300–400 mesh silica gels. Ultrapure water was produced by a Milli-Q Direct 16 system of Millipore. UV-Vis absorption spectra were gained on a Shimadzu UV-2550 spectrophotometer (Shimadzu, Kyoto, Japan). Fluorescence spectra were obtained on a Cary Eclipse fluorescence spectrophotometer from Agilent. ^1^H- and ^13^C-NMR spectra were recorded with a Mercury plus instrument at 400 and 100 MHz by using DMSO-*d_6_* as the solvents. MS spectra were recorded on a MALDI-TOF MS Performance (Shimadzu, Japan).

### 2.2. Molecular Synthesis

**Figure 1:**
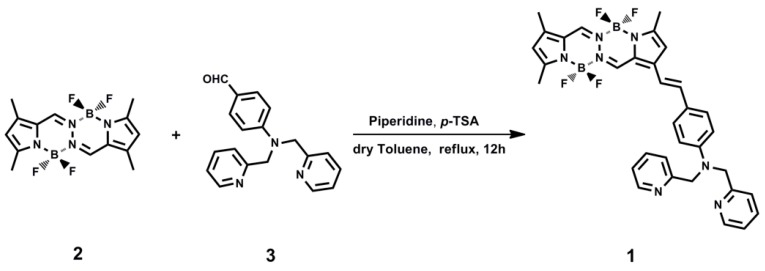
The synthesis route of **1**.

Compound **2** [30] and **3** [29] were synthesized according to literatures, while **1** was synthesized as illustrated in Figure 1. Dry toluene used in synthesis was distilled over sodium and benzophenone. A mixture of **2** (0.50 g, 1.48 mmol), **3** (0.45 g, 1.48 mmol), and *p*-toluenesulfonicacid (1 g, 5.81 mmol) was dissolved in dry toluene (50 mL), followed by the addition of 1 mL piperidine as catalyst. The mixture was refluxed with stirring for 12 h under an atmosphere of nitrogen, during which time the color of the reaction mixture changed from pale yellow to red. After cooling to room temperature, the mixture was poured into H_2_O (100 mL) and extracted with CH_2_Cl_2_. After solvent removal, the crude product was purified by column chromatography (silica gel, CH_2_Cl_2_/petroleum ether, *v*/*v* = 2/1), producing a dark purple solid (0.45 g), yield 49%. ^1^H-NMR (400 MHz, DMSO-*d_6_*) δ = 8.61–8.60 (m, 2H), 7.92 (s, 1H), 7.84 (s, 1H), 7.65–7.62 (m, 2H), 7.38 (d, *J* = 5.6 Hz, 2H), 7.25 (d, *J* = 5.2 Hz, *J* = 5.2 Hz, 2H), 7.20–7.18 (m, 2H), 7.17 (d, *J* = 2.8 Hz, 2H), 6.72 (d, *J* = 6.0 Hz, 2H), 6.68 (s, 1H), 6.16 (s, 1H), 4.87 (s, 4H), 2.48 (s, 3H), 2.32 (s, 3H), 2.31 (s, 3H). (Figure S1, Supplementary Materials). ^13^C-NMR (100 MHz, DMSO-*d_6_*) δ = 157.67, 150.75, 150.07, 149.40, 148.72, 140.24, 139.84, 136.69, 136.49, 133.39, 132.27, 128.63, 124.96, 124.08, 122.98, 121.81, 120.34, 117.88, 114.17, 113.24, 112.24, 56.83, 13.66, 10.73, 10.65. (Figure S2, Supplementary Materials). MALDI-TOFMS: *m*/*z* calculated for C_33_H_31_B_2_F_4_N_7_: 623.28; found: 623.47. (Figure S3, Supplementary Materials).

### 2.3. Sample Preparation and Spectral Measurements

A stock solution (0.5 mM) of sensor **1** was prepared in acetonitrile. Metal ion solutions of Cd^2+^, Zn^2+^, Mn^2+^, Pb^2+^, Cu^2+^, Co^2+^, Mg^2+^, Ca^2+^, Ba^2+^, Fe^2+^, and Hg^2+^ were prepared by dissolving the corresponding nitrate salts in acetonitrile. These stock solutions were diluted to needed concentrations for sensor testing. UV-Vis and fluorescent spectra were measured under room temperature. Briefly, 2.5 mL solution of **1** (2 μM) was put into a 1 cm quartz cuvette, followed by addition of different concentrations of metal ion. The series of concentrations of metal ions were thus added and were measured for the absorption and fluorescence spectra. Since added volume of the metal ion stock solution was small (up to 8 μL), the concentration of sensor **1** would remain almost unchanged. For fluorescence spectra measurement, the excitation wavelength was set at 410 nm and slit widths at 5 nm/10 nm.

## 3. Results and Discussion

### 3.1. Spectral Change of **1** Upon Titration with Cd^2+^

**Figure 2:**
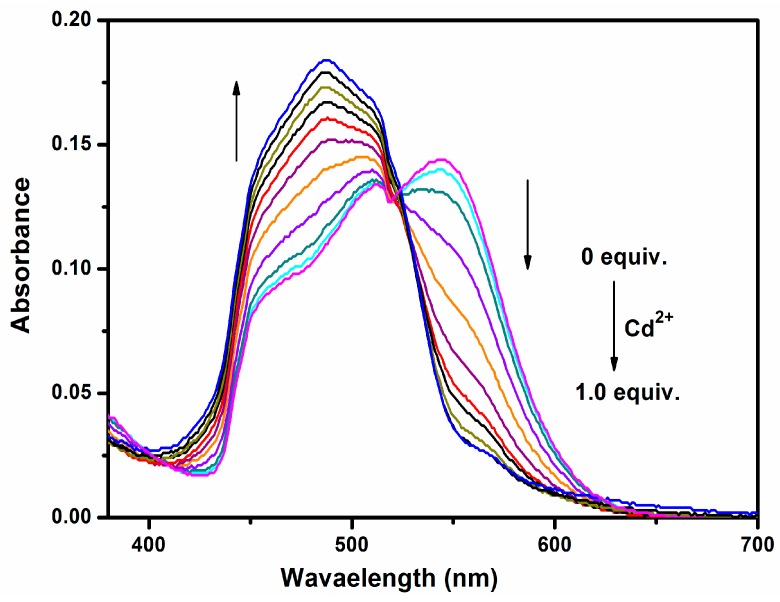
UV-vis absorption spectral change recorded for an acetonitrile solution of sensor **1** (2 μM) upon the titration of Cd^2+^ ion.

**Scheme 2:**
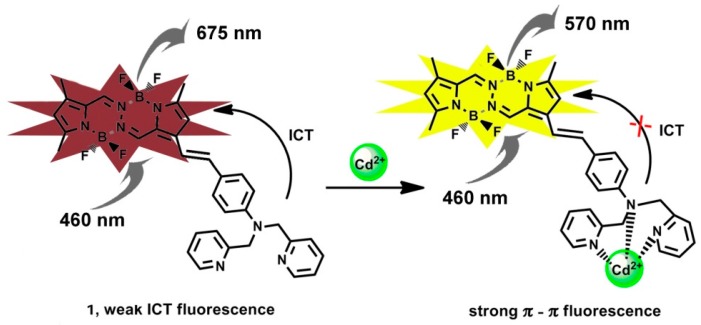
Sensing mechanism of **1** towards Cd^2+^.

As shown in Figure 2, the absorption spectrum of pristine **1** has two pronounced peaks around 505 nm and 550 nm. These two absorption peaks are significantly red-shifted in comparison with those of Me_4_BOPHY, which has the corresponding two peaks at 444 nm and 467 nm. Such spectral redshift is due to the ICT electronic transition, as previously observed in other electron donor-acceptor molecules [29]. In molecule **1** the fluorophore Me_4_BOPHY is in full conjugation with the aniline group of BPA through the vinyl bridge (Scheme 1), thus facilitating the ICT transition. Upon titration with Cd^2+^ ion, the absorption at 550 nm gradually decreased, accompanied by a rising blue-shifted absorption peak centered at 475 nm. An isosbestic point was clearly seen around 520 nm, indicating the stoichiometric conversion of molecule **1** from unbound to the Cd^2+^-bound state. As the concentration of Cd^2+^ increased, the color of the solution turned from red to bright yellow, consistent the absorption spectral change shown in Figure 2. The observed spectral change is due to the binding of Cd^2+^ at the BPA chelator (Scheme 2), which in turn reduces the electron-donating capability of the aniline moiety. As a result, the ICT transition of molecule **1** is diminished. Indeed, as molecule **1** is fully chelated, the absorption spectrum becomes mostly characteristic of the π − π transition of the Me_4_BOPHY part, centered around 475 nm (Figure 2).

**Figure 3:**
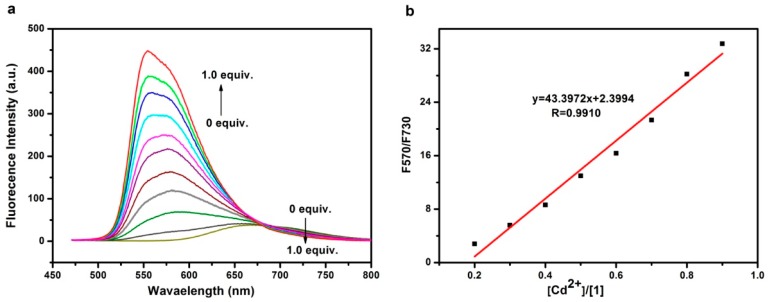
(**a**) Fluorescence spectral change recorded for an acetonitrile solution of sensor **1** (2 μM) upon titration of Cd^2+^ ion; (**b**) The ratio of fluorescence intensity (*F570/F730*) measured for the same solutions at 570 nm and 730 nm as a function of the concentration of Cd^2+^ (relative to that of **1**), showing linear fitting as indicated in the plot.

The same series of titration of Figure 2 was also monitored for fluorescence spectral change, as shown in Figure 3a. The unbound molecule **1** has an emission band centered at 675 nm, which is significantly red-shifted in comparison to the two emission bands (485 nm and 518 nm) that are typically observed for the fluorophore of tetramethyl substituted BOPHY (Me_4_BOPHY). The strong redshift is mainly a result of the ICT transition (Scheme 2), which in turn is caused by the BPA substitution. Upon binding with the Cd^2+^ ion, the emission peak was blue-shifted to 570 nm, implying that the ICT transition is diminished, as discussed above. The fluorescence quantum yield of pristine **1** determined as 7.6% by using Rhodamine B in acetonitrile as a standard (φ_F_ = 0.89, λ_ex_ = 495 nm). By comparing the total fluorescence intensity and the absorbance at the same excitation wavelength 495 nm between the unbound and Cd^2+^-bound state of **1**, the fluorescence quantum yield of Cd^2+^-bound **1** can be estimated to be 44.2%. The spectral change shown in Figure 3a enables ratiometric sensing by plotting the ratio of fluorescent intensity at 570 nm and 730 nm (*F570/F730*) as a function of the concentration of Cd^2+^ (relative to that of **1**), as shown in Figure 3b. An approximately linear relationship was obtained, allowing for determining the concentration of Cd^2+^ using this linear calibration. The limit of detection (LOD) can be projected by taking three times the standard deviation of measurement as the detectable signal, that is, 0.3 in this study. Using the slope of the linear fitting of Figure 3b, we can determine the LOD to be 6.9 nM, or 0.77 ppb, which is far below the safety value set for drinking water by WHO (3 ppb), indicating a strong feasibility of using sensor **1** for trace level detection of Cd^2+^. The ratiometric sensing, relying on the fluorescence measurement of both bound and unbound state of **1**, could potentially improve the robustness of signal by canceling the interference from the environment. By comparing with other fluorescence sensors for Cd^2+^ reported in literature (Table 1), sensor **1** developed in this study has many advantages over other Cd^2+^ sensors.

### 3.2. Sensing Mechanism and Job’s Plot

**Figure 4:**
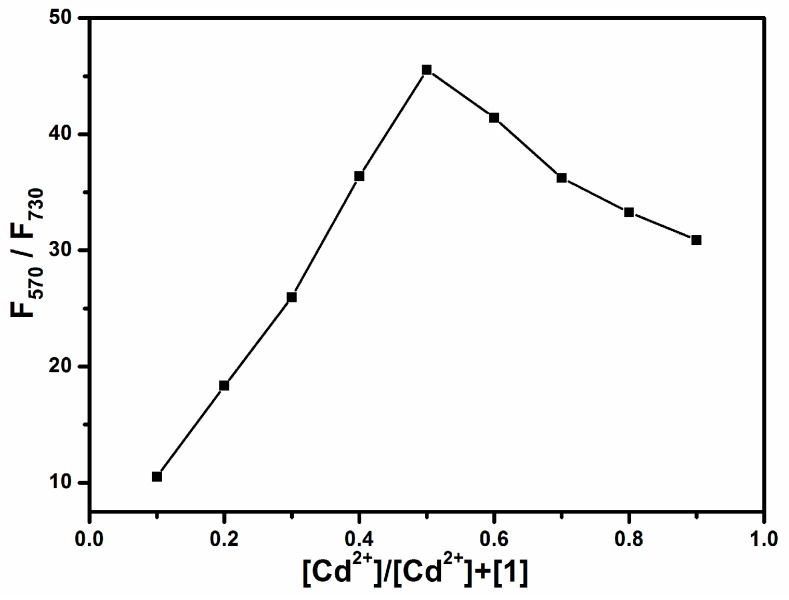
Job’s plot of the binding between **1** and Cd^2+^ in acetonitrile, with the total concentration of the two species fixed at 2 μM.

As illustrated in Scheme 2, the sensing mechanism of **1** relies on switching the fluorescence from ICT transition to local π − π transition at the BOPHY site. The BPA chelator affords strong binding to the Cd^2+^ ion, and this weakens the electron donating power of the aniline moiety, thus diminishing the ICT transition. The tridentate chelation of BPA forms 1:1 complex with Cd^2+^ ion, as also reported in other studies wherein the same chelator was used [29]. The 1:1 chelation stoichiometry was also confirmed in this study through a Job’s plot approach [44], as shown in Figure 4. Job’s plot is commonly used to determine the stoichiometry of a complex between two species, for which the total molar concentrations of the two species (here molecule **1** and Cd^2+^ ion) are kept constant, while their relative concentrations are varied. A measured variable (here the fluorescence intensity ratio, *F570/F730*) that is dependent on the complex formation can be plotted as a function of the molar fractions of the binding species. The maximum of the plot corresponds to the stoichiometry of the complex formed. In this study, the total concentration of molecule **1** and Cd^2+^ ion was fixed at 2 μM, and the molar ratio of the two species was changed from 1:9 to 9:1, and the fluorescence intensity ratio *F570/F730* was measured under the same conditions. Clearly, as shown in Figure 4, the maximum of the plot corresponds to a 1:1 complex between **1** and Cd^2+^. 

### 3.3. Sensing Selectivity 

**Figure 5:**
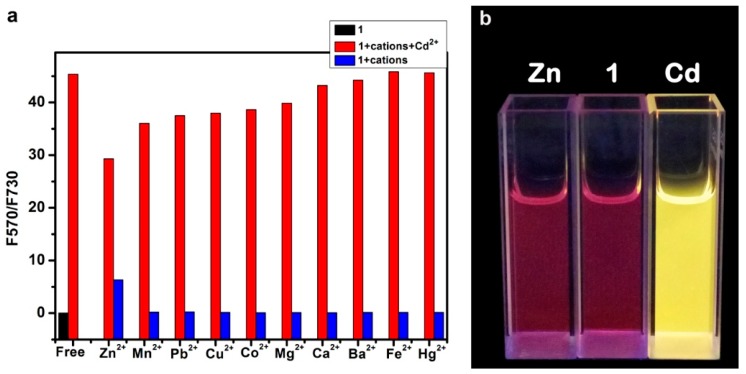
(**a**) Fluorescence intensity ratio (*F570/F730*) measured for sensor **1** in acetonitrile (2 μM) in the absence of metal ions (black), and in the presence of various metal ions (2 μM), (blue), followed by addition of 2 μM Cd^2+^ into each of the eleven solutions (red); (**b**) Photographs taken for the 2 μM solution of **1**, in comparison to the ones containing 2 μM of Zn^2+^ and Cd^2+^.

The high selectivity of **1** towards Cd^2+^ ion was examined by comparative experiments, which were conducted by repeating the same fluorescence measurements shown in Figure 3 but in the presence of 10 other common metal ions, Mn^2+^, Pb^2+^, Cu^2+^, Co^2+^, Mg^2+^, Ca^2+^, Ba^2+^, Fe^2+^, Hg^2+^, and Zn^2+^. In contrast to the efficient spectral change observed for Cd^2+^ (far left bar in the figure), all of the other metal ions (except for Zn^2+^) demonstrated almost no spectral change, as indicated by the low values of *F570/F730* measured under the same experimental conditions (Figure 5a). However, upon the addition of Cd^2+^ ion at the same concentration, all of the 10 solutions containing the different metal ions showed dramatic fluorescence change at the same degree as that observed for the solution of **1** + Cd^2+^. This observation indicates good sensing selectivity for molecule **1** towards Cd^2+^, which in turn is largely due to the strong chelation, as illustrated in Scheme 2. The mild fluorescence response observed for Zn^2+^ ion is not surprised considering the similar coordination property between Zn^2+^ and Cd^2+^. However, due to the weaker electron affinity of Zn^2+^ ion (with standard reduction potential of −0.7 V, as compared to that of Cd^2+^, −0.4 V), the binding with Zn^2+^ cannot block the ICT transition as effectively as Cd^2+^. Indeed, as shown in Figure 5b, under the same concentration the solution of **1** + Cd^2+^ exhibited a dramatic fluorescence color change (consistent with the spectral measurement shown Figure 3), whereas the solution of **1** + Zn^2+^ remained about the same color as the solution of **1**. Such dramatic difference in color change provides additional feature to distinguish Cd^2+^ from other metal ions when using **1** as sensor. 

### 3.4. Fast Sensor Response

**Figure 6:**
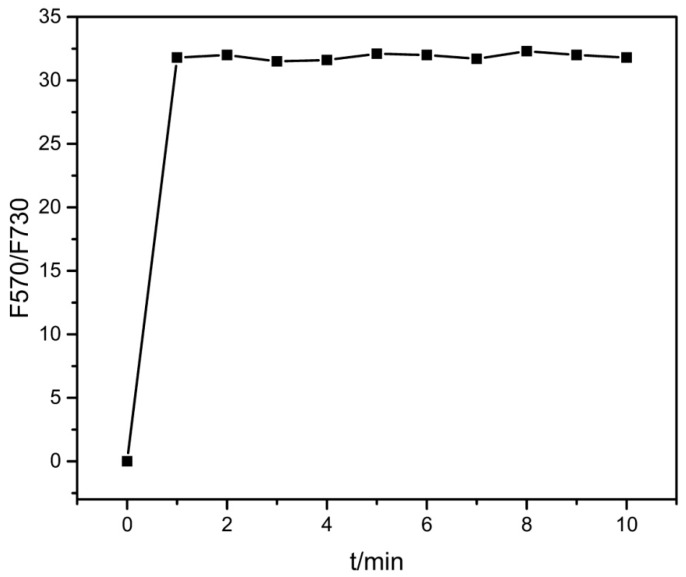
Time course of the fluorescence intensity ratio (*F570/F730*) change measured on an acetonitrile solution of sensor **1** (2 μM) upon addition of Cd^2+^ ion (2 μM).

The sensor **1** could rapidly detect Cd^2+^ ion, as shown in Figure 6. When we put 2 μM Cd^2+^ into 2 μM sensor **1** solution, the fluorescence intensity ratio (*F570/F730*) of sensor **1** quickly increased and reached a stable value within 1 min. This is a good trait for fast and real-time determination. 

In addition to the high sensitivity and selectivity observed above, sensor **1** also demonstrated a fast response, consistent with the strong chelation with Cd^2+^. As shown in Figure 6, the ratiometric fluorescence response of **1** was finished in one min upon addition of 1:1 Cd^2+^ ion. Due to the experimental operation limit, we could not monitor the sensor response in any faster time scale, though the real response time of **1** seems to be in seconds or even faster. This fast fluorescence response makes sensor **1** ideal for real-time monitoring, particularly for in-field detection. Also indicated from Figure 6 is the high photostability sensor **1**, wherein the fluorescence of **1** was measured ten times after binding with Cd^2+^ ion, but no significant change in the fluorescence intensity was observed. 

## 4. Conclusions

We report on a novel fluorescence sensor **1** for the selective detection of Cd^2+^ ion with LOD down to 0.77 ppb. The sensor molecule is based on a fluorophore of Me_4_BOPHY in conjugation with an electron donor group, namely BPA, which also affords strong binding with Cd^2+^. The electron donor-acceptor conjugation enables ICT fluorescence at long wavelength, desired for sensor development. Upon binding with the Cd^2+^ ion, the fluorescence is switched from ICT transition to be the π − π transition, which dominated by the Me_4_BOPHY fluorophore, which is located in much shorter wavelength region. Such dramatic fluorescence change enables ratiometric sensing by measuring the relative emission intensity at the two wavelengths as a function of the concentration of Cd^2+^ ion, thus allowing for quantitative detection of Cd^2+^. High selectivity towards Cd^2+^ was also evidenced for the sensor as examined with ten other common metal ions.

## Figures and Tables

**Table 1:** The comparison of **1** with other Cd^2+^ sensors in literature.

Refs.	LOD (mol L^−1^)	Wavelength of Emission	Solvent Used
[7]	1.97 × 10^−7^	456 nm	CH_2_Cl_2_/CH_3_CN (1/9)
[8]	2.76 × 10^−7^	500 nm	H_2_O
[18]	1.76 × 10^−7^	495 nm/558 nm	HEPES
[29]	Not available	597 nm	CH_3_COCH_3_/H_2_O (9/1)
This work	6.9 × 10^−9^	570 nm/730 nm	CH_3_CN

## Supplementary Materials

The Supplementary Materials are available online at http://www.mdpi.com/1424-8220/17/11/2517/s1.
