# Supplementary material for: A Ratiometric Fluorescent Sensor for Cd2+ Based on Internal Charge Transfer
Source: Sensors (Basel). 2017 Nov 2;17(11):2517. doi: 10.3390/s17112517 (PMC5713183; doi:10.3390/s17112517)
Supplement: Supplementary file 1 [file sensors-17-02517-s001.pdf]

Supplementary Material

A ratiometric fluorescent sensor for Cd^{2+} based on internal charge transfer

Dandan Cheng[1, †], Xingliang Liu[1, †], Yadian Xie[1], Haitang Lv[1], Zhaoqian Wang[1], Hongzhi Yang[1], Aixia Han[1, 2,*], Xiaomei Yang[2], Ling Zang[2,*]

[1] Chemical Engineering College, Qinghai University, Xining 810016, China; 1061643811@qq.com(D.C.); liuxingliang@qhu.edu.cn(X.L.); 1126867541@qq.com(Y.X.); 550538500@qq.com(H.L.); 502430180@qq.com(Z.W.); 1033485623@qq.com(H.Y.)

[2] *Department of Materials Science and Engineering, University of Utah, Salt Lake City, Utah 84108, USA.* jaimee@eng.utah.edu(X.Y.)

[†]These two authors contributed equally.

[*]Correspondence: hanaixia@tsinghua.org.cn(A.H.); Tel.: +86-971-5310-427
lzang@eng.utah.edu (L. Z.); Tel.: +1-801-587-1551

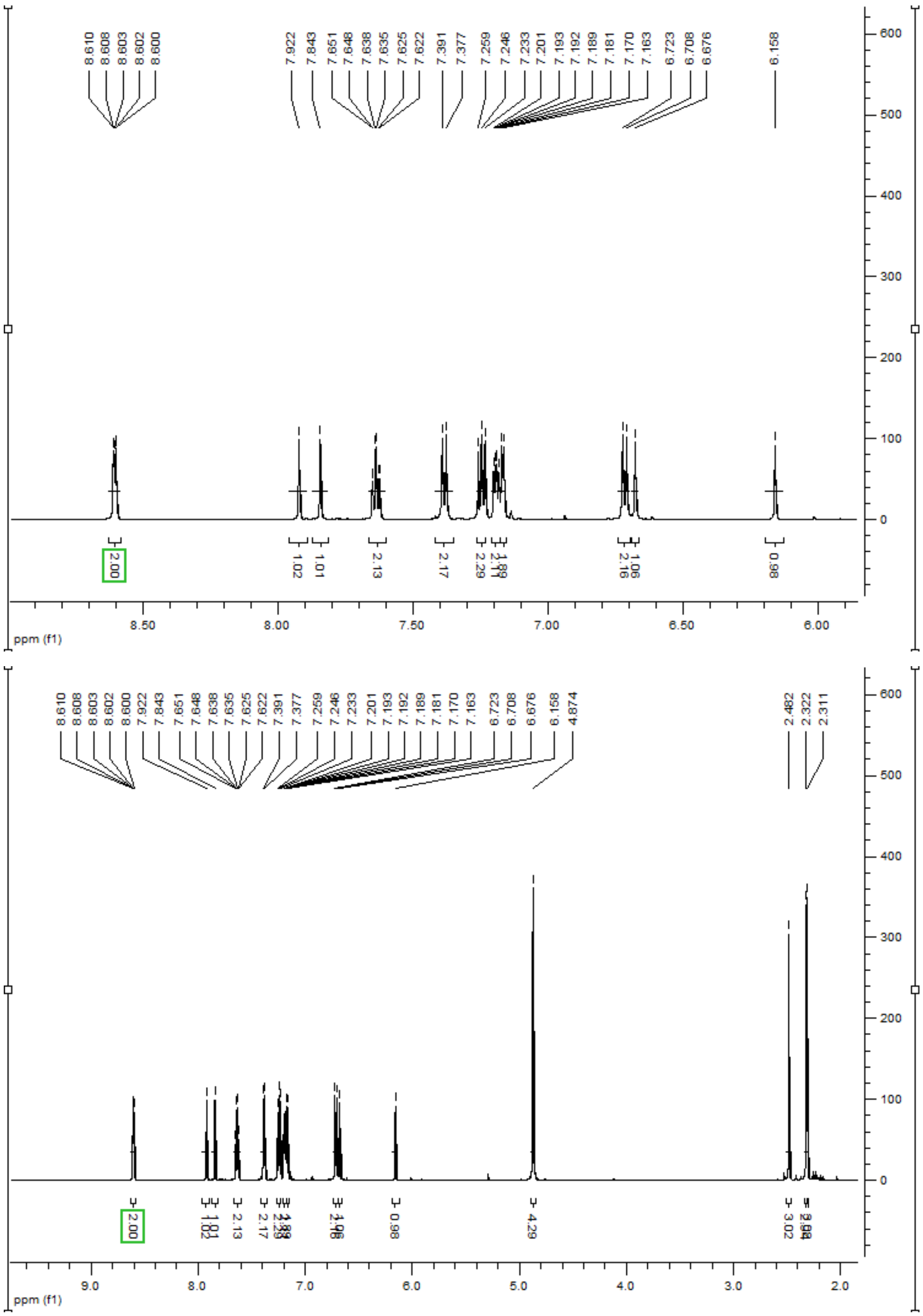

Fig.S1. ^{1}H NMR (400 MHz) spectrum of sensor **1** in DMSO-d_6.

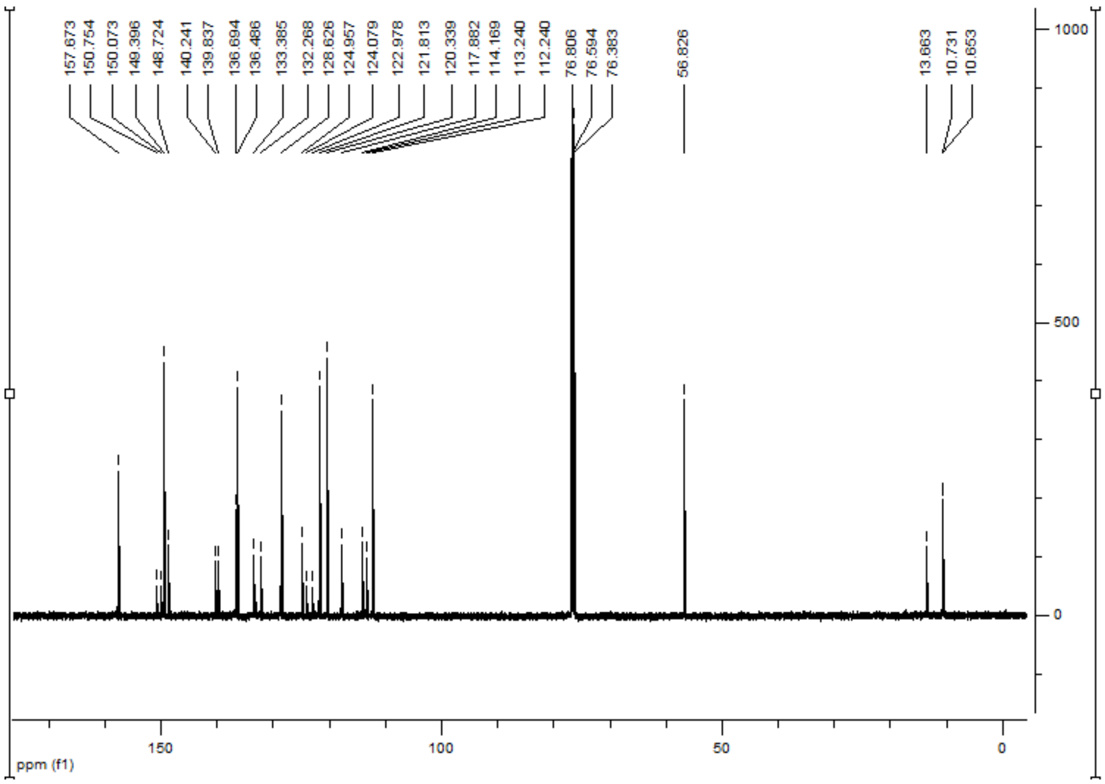

Fig. S2. ^{13}C NMR (100 MHz) spectrum of sensor **1** in DMSO-d_6.

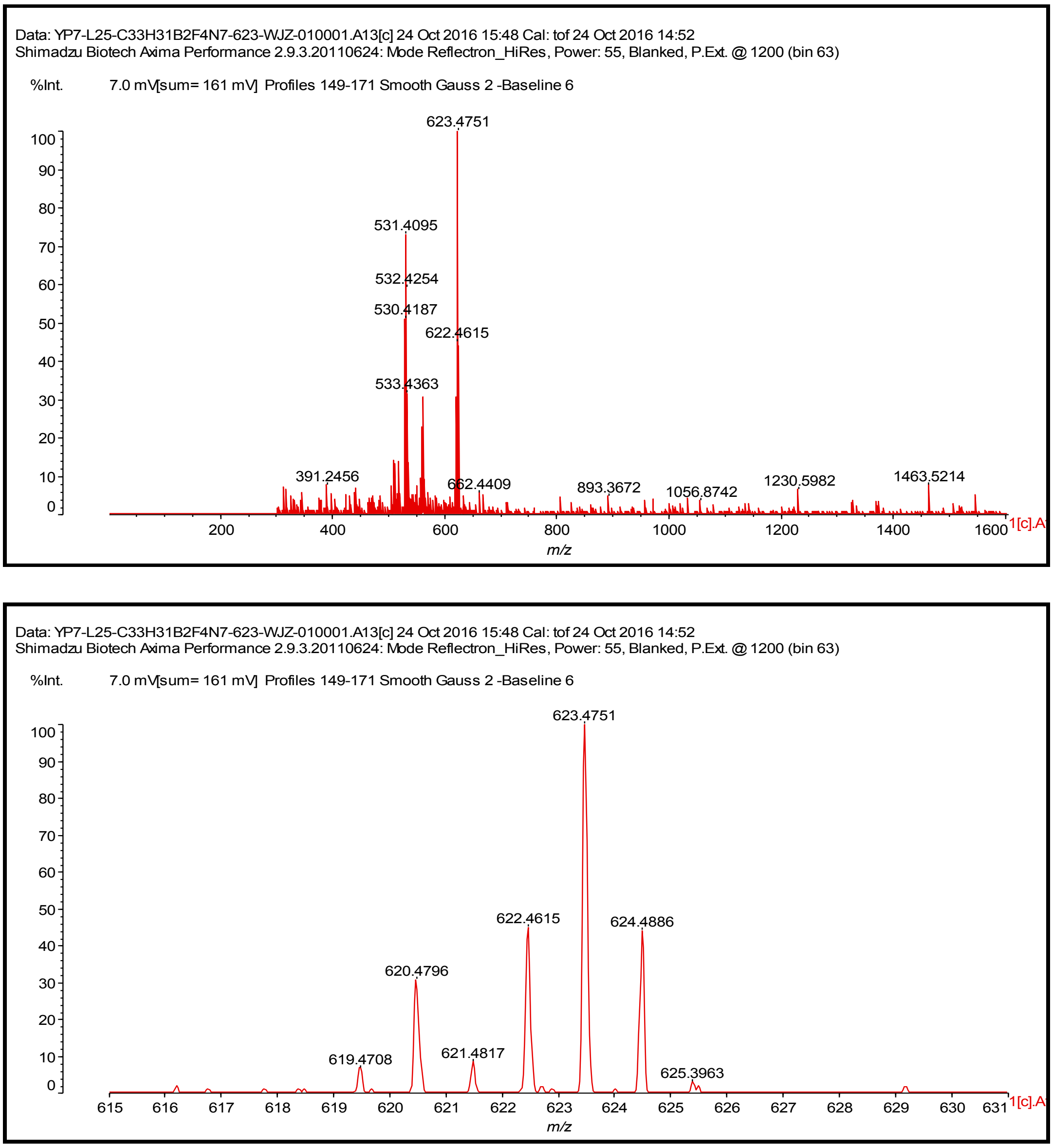

Fig. S3. MALDI/TOF MS spectrum of sensor **1**.
